# Identification and discrimination of *Toxoplasma gondii*, *Sarcocystis* spp., *Neospora* spp., and *Cryptosporidium* spp. by righ-resolution melting analysis

**DOI:** 10.1371/journal.pone.0174168

**Published:** 2017-03-27

**Authors:** Hllytchaikra Ferraz Fehlberg, Bianca Mendes Maciel, George Rêgo Albuquerque

**Affiliations:** 1 Graduation program in Animal Science, Santa Cruz State University, Ilhéus (BA), Brazil; 2 Department of Biological Sciences, Santa Cruz State University, Ilhéus (BA), Brazil; 3 Department of Agricultural and Environmental Sciences, Santa Cruz State University, Ilhéus (BA), Brazil; Texas A&M University College Station, UNITED STATES

## Abstract

The objective of this study was to standardize the high-resolution melting method for identification and discrimination of *Toxoplasma gondii*, *Sarcocystis* spp., *Neospora* spp., and *Cryptosporidium* spp. by amplification of 18S ribosomal DNA (rDNA) using a single primer pair. The analyses were performed on individual reactions (containing DNA from a single species of a protozoan), on duplex reactions (containing DNA from two species of protozoa in each reaction), and on a multiplex reaction (containing DNA of four parasites in a single reaction). The proposed method allowed us to identify and discriminate the four species by analyzing the derivative, normalized, and difference melting curves, with high reproducibility among and within the experiments, as demonstrated by low coefficients of variation (less than 2.2% and 2.0%, respectively). This is the first study where this method is used for discrimination of these four species of protozoa in a single reaction.

## Introduction

Coccidia are in the phylum Apicomplexa of Protozoa and are well distributed worldwide [1]. They are responsible for important diseases with worldwide epidemiological and economic relevance for both human and animal health. Among these diseases, we highlight neosporosis (caused by *Neospora* spp.), toxoplasmosis (*Toxoplasma gondii*), cryptosporidiosis (*Cryptosporidium* spp.), and sarcocystosis (*Sarcocystis* spp.) [1–2].

*T*. *gondii*, *N*. *caninum*, and *S*. *neurona* are members of the Sarcocystidae family that can infect a wide variety of intermediate hosts, causing severe neurological disorders in animals [3–8]. Reproductive problems, including abortion, are also consequences of *T*. *gondii* and *N*. *caninum* infections [6, 9]. *Cryptosporidium parvum* causes serious intestinal disorders, triggering diarrheal syndrome in humans and animals, especially in immunocompromised individuals and may lead to a lethal acute phase [10–11].

Diseases associated with these species may be underdetected due diagnostic challenges related to detection and identification of oocysts in fecal samples by traditional microscopic and morphological techniques, which are not reliable for species identification [12]. Thus, the molecular technique of polymerase chain reaction (PCR) has been used for the diagnosis of these protozoa owing to its specificity and sensitivity and because it can be performed rapidly [13–14]. However, discrimination of various coccidia by PCR requires more than one primer pair in specific reactions for each parasite; this situation makes this technique laborious. For disease outbreak investigations, a screening methodology that enables discrimination of coccidia in a single reaction would have a big advantage because rapid and correct diagnosis allows an early intervention to control the disease. In this context, high-resolution melting (HRM) may be a reliable alternative.

HRM is a post-real-time PCR qualitative molecular method that is capable of discriminating DNA sequences based on their composition, size, G/C content, or complementarity of strands [15]. It may also identify and discriminate various infectious agents by melting curves [16–17] because amplicons of different sequences may be distinguished on the basis of the curve shape even when they share the same melting temperature (Tm). In this technique, the use of saturation dyes enables detection of Tm differences as small as 0.01°C during the acquisition of fluorescence [18]. It is a relatively new technology and has been used for various purposes, such as discrimination studies and genotyping of organisms [15, 19], detection of virulence genes and analysis of antibiotic resistance [20], tracking of mutations, and identification of single nucleotide polymorphisms (SNPs) of homozygous and heterozygous genotypes [21] or the percentage of methylation [22].

The aim of this study was is to develop a rapid, sensitive and discriminatory diagnostic test for *T*. *gondii*, *Sarcocystis* spp., *Neospora* spp. and *Cryptosporidium* spp. using a single primer pair targeting 18S ribosomal DNA (rDNA) in a single reaction.

## Material and methods

### Strains of coccidia and DNA extraction

*T*. *gondii* strains RH (Type I), PTG (Type II), and CTG (Type III) from the peritoneal fluid of albino mice after tachyzoites inoculation; the *S*. *neurona* 37-R strain from naturally infected possums [23]; the *N*. *caninum* NcBA strain isolated from the brain of a dog with neosporosis [24]; and *C*. *parvum* 13O, 13F, and 13B isolates from coccidia-positive fecal samples of dogs were used in this study. *T*. *gondii* CTG and PTG strains were kindly provided by Prof. Dr. Hilda Pena (University of São Paulo—USP), *S*. *neurona* 37-R and *N*. *caninum* were kindly provided by Prof. Fernando Pita Gondim (Federal University of Bahia—UFBA), and *C*. *parvum* (13O, 13F, and 13B isolates) were kindly provided by Prof. Aristeu Vieira da Silva (Feira de Santana State University—UEFS). *C*. *parvum* DNA was extracted using the QIAamp DNA Stool Mini Kit (Qiagen, Hilden, Germany), and the DNA of other parasites was extracted using the EasyDNA Kit (Invitrogen, Carlsbad, CA, USA). The DNA was quantified on a NanoDrop 2000 (Thermo Scientific, Waltham, MA, USA), then diluted to 20 ng/μL, and stored at −20°C. All isolated DNA samples were used to standardize the HRM technique.

### Design of primers

The forward primer 5´-GTTGTTGCAGTTAAAAAGCTGGT-3´ and reverse primer 5´-ATCTAAGAATTTCACCTCTGACAGT-3´ were designed in the primer Express Software, v3.0 (Applied Biosystems, Life Technologies, Carlsbad, CA, USA), using as a template the 18S rDNA sequences of various coccidia available in the GenBank database, such as *T*. *gondii* (gb EF472967.1), *S*. *hominis* (AF176945 gb), *N*. *caninum* (U16159.1 gb), *C*. *parvum* (gb AF093494.1), *Hammondia hammondi* (AF096498 gb. 1), *Cyclospora cayetanensis* (AF111183.1), *Cystoisospora timoni* (AY279205.1), *Nephroisospora eptesici* (gb EU334134.1), and *Isospora belli* (gb AF106935.1). The amplification of target sequences was intended to produce a ~320-bp amplicon containing conserved regions flanking sites with genetic variability among the different coccidia (S1 File).

### PCR

Initially, conventional PCR was performed using genomic DNA from each coccidian, and the same pair of primers was used. The assay was conducted on a thermocycler Proflex PCR system (Applied Biosystems). Each 50-μL PCR reaction contained 1× PCR buffer (Invitrogen), 2.5 mM MgCl_2_ (Invitrogen), 0.2 μM dNTP mix, 0.2 μM each primer (Invitrogen), 2 U of Taq DNA polymerase (Invitrogen), and 5 μL of DNA. Sterile nuclease-free water was added to 50 μL. The amplification consisted of: 10 min at 95°C for initial denaturation followed with 40 cycles at 95°C for 15 sec, 60°C for 1 min, and 72°C for 10 sec; and a final extension step of 10 min at 72°C. The PCR products were analyzed by electrophoresis in a 1% agarose gel stained with SYBR-Safe (Invitrogen) and examined under ultraviolet light. Subsequently, the product was purified using the PureLink® Genomic DNA Kit (Invitrogen), quantified on a NanoDrop 2000 (Thermo Scientific, USA), and diluted to 20 ng/μL for later use in the HRM assay.

### HRM

The HRM analyses were performed on an Applied Biosystems 7500 Fast (Life Technologies). All the assays were carried out in duplicate, in a uniplex reaction (containing DNA from a single species of a protozoan), a duplex reaction (containing DNA from two species of protozoa in each reaction: *T*. *gondii + N*. *caninum*, *T*. *gondii + S*. *neurona*, *T*. *gondii + C*. *parvum*, *N*. *caninum + S*. *neurona*, *N*. *caninum + C*. *parvum*, or *C*. *parvum + S*. *neurona*), or a multiplex reaction (containing DNA of the four parasites in a single reaction). For these assays, we used the MeltDoctor^TM^ HRM Master Mix Kit (Applied Biosystems, USA) in a final volume of 20 μL containing 1× MeltDoctor^TM^ HRM Master Mix, 0.3 μM each primer, and 40 ng of genomic DNA (or 40 ng of the purified PCR product). The amplification reactions were conducted under the following conditions: initial denaturation at 95°C for 10 minutes, followed by 40 cycles of 95°C for 15 seconds and 60°C for 1 minute. A melting curve at 95°C for 20 seconds, 95°C for 3 seconds, and 60°C for 30 seconds was recorded after each reaction. The reactions were analyzed by High Resolution Melting (HRM) Software v3.01 (Thermo Fisher Scientific), assessing differences in melting curve shapes and generating three types of graphs represented by derivative, normalized, and difference curves.

### Reproducibility of HRM

The intra- and interexperiment reproducibility was assessed by calculating the averages, standard deviations, and coefficients of variation of Tm peaks generated by the HRM software. To assess the intraexperiment reproducibility, Tm of triplicates was analyzed for the same reaction. The interexperimental variation was assessed in three different reactions, including three repetitions of the same sample.

### Sequencing of the 18S rDNA amplification product

The PCR products (approximately 320 bp) of each protozoan were purified with the PureLink® Genomic DNA Kit (Invitrogen) and sent to Ludwig Biotec (Rio Grande do Sul, Brazil) for sequencing. Sequencing was performed on the *ABI-PRISM* 3100 Genetic Analyzer platform (Applied Biosystems) in both directions in duplicate. The chromatograms were analyzed in the Phred software. Sequences were assembled into contigs using the CAP3 software, aligned in ClustalW (version 1.83), and manually corrected using the BioEdit Sequence Alignment Editor. Then, the sequences were compared to each other to find genetic variability sites in the amplicons of 18S rDNA obtained by the primer pair described in this study. The sequences were also aligned by the BLASTn software (https://blast.ncbi.nlm.nih.gov/) to assess the degree of similarity of the amplicons with the sequences available in databases.

## Results and discussion

This is the first study on identification and discrimination of *T*. *gondii*, *Cryptosporidium* spp., *Sarcocystis* spp., and *Neospora* spp. by HRM using a single pair of primers targeting 18S rDNA. This method was not tested for its ability to differentiate other species into the genera *Neospora*, *Cryptosporidium* and *Sarcocystis*. Other studies using this method have addressed the detection of *T*. *gondii* [25–26] and *C*. *parvum* [27]. The detection and discrimination of coccidia (*Sarcocystis*, *Cryptosporidium*, *Eimeria*, *Isospora*, and *Toxoplasma*) have already been performed using real-time PCR followed by melting curve analysis [12], but those authors used a cocktail of seven different primer pairs. No articles were found where HRM was used for detection of *Neospora* spp.

**Fig 1:**
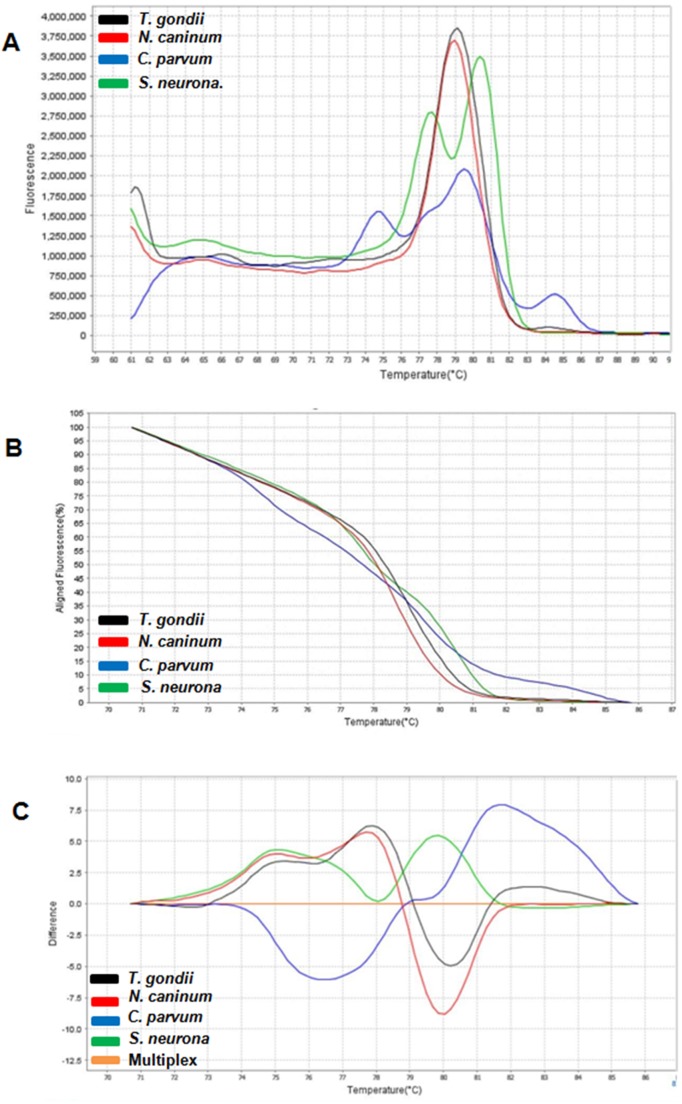
**Derivative melting curve (A), normalized melting curve (B), and difference melting curve (C) for coccidian**. *Toxoplasma gondii* (black), *Neospora caninum* (red), *Cryptosporidium parvum* (blue), *Sarcocystis neurona* (green) and multiplex reaction containing DNA from all four protozoa (orange).

**Fig 2:**
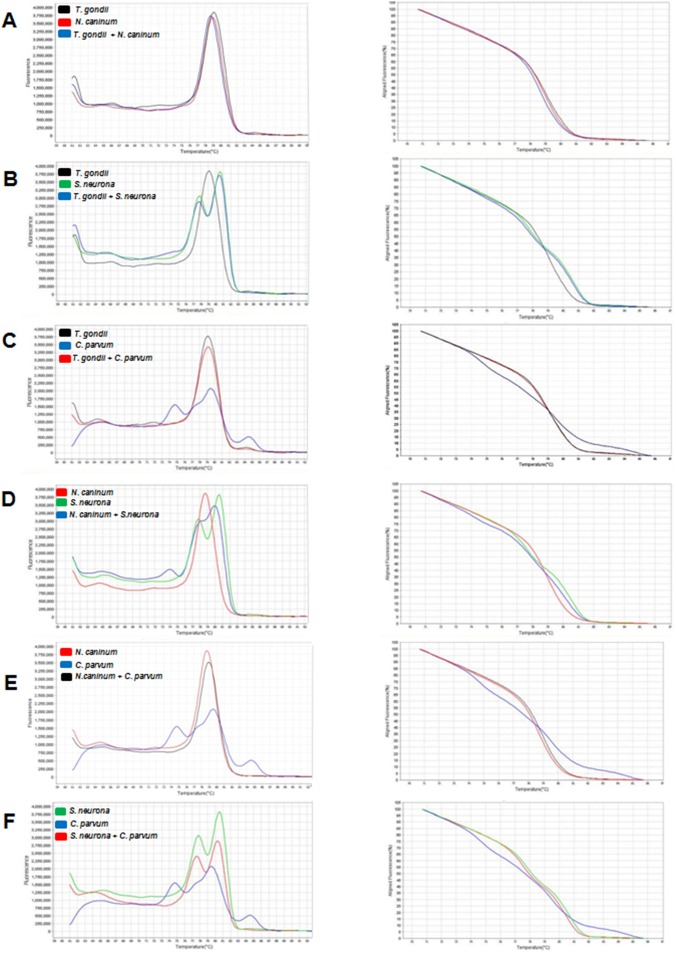
Derivative melting curve (left) and normalized melting curve (right) of HRM duplex assays. (A) *Toxoplasma gondii* and *Neospora caninum*; (B) *T*. *gondii* and *Sarcocystis neurona*; (C) *T*. *gondii* and *Cryptosporidium parvum*; (D) *N*. *caninum* and *S*. *neurona*; (E) *N*. *caninum* and *C*. *parvum*; and (F) *S*. *neurona* and *C*. *parvum*.

The patterns of the derivative, normalized, and difference melting curves were consistent for each distinguishable species, both in individual assays and in combined assays (Figs 1 and 2). The melting curves showed characteristic Tm peaks of each test, with one, two, or three peaks, depending on the parasite (Table 1). The evaluation of inter- and intraexperiment reproducibility yielded a low coefficient of variation: less than 2.2% and 2%, respectively (Table 1), pointing to high reproducibility of the assay.

**Table 1:** Intra-assay and inter-assay reproducibility of the melting temperatures (Tm) produced by HRM during the analysis to differentiate the coccidia *Toxoplasma gondii*, *Sarcocystis neurona*, *Neospora caninum* and *Cryptosporidium parvum*.

		Intra-assay reproducibility ^a^			Inter-assay reproducibility ^b^		
Analysis	Protozoan	Tm 1	Tm 2	Tm 3	Tm 1	Tm 2	Tm 3
Individual	*T*. *gondii*	79.85 (± 0.64)	__	__	79.50 (±1.30)	__	__
		CV = 0.8%			CV 1.6%		
	*N*. *caninum*	79.35 (±1.34)	__	__	79.16 (± 1.47)	__	__
		CV = 1.7%			CV = 1.9%		
	*S*. *neurona*	78.15 (±1.34)	81.00 (±1.27)	__	78.03 (± 1.28)	80.79 (± 1.38)	__
		CV = 1.7%	CV = 1.6%		CV = 1.6%	CV = 2%	
	*C*. *parvum*	76.65 (±1.34)	80.7 (±1.27)	85.15 (± 1.34)	76.03 (±1.33)	80.74 (± 1.33)	84.78(±1.33)
		CV = 1.7%	CV = 1.5%	CV = 1.6%	CV = 1.8	CV = 2%	CV = 2%
Duplex	*T*. *gondii* +	78.75 (±1.34)	__	__	78.91 (±1.36)	__	__
	*N*. *caninum*	CV = 1.7%			CV = 1.7%		
	*T*. *gondii +*	79.05 (±1.77)	__	__	78.9 (±1.38)	__	__
	*S*. *neurona*	CV = 2.2%			CV = 1.7%		
	*T*. *gondii +*	78.55 (±1.34)	85.4 (±1.27)	__	78.95 (±1.27)	83.5 (±1.27)	__
	*C*. *parvum*.	CV = 1.7%	CV = 1.5%		CV = 1.6%	CV = 2%	
	*N*. *caninum +*	76.8 (±1.27)	79.4 (±1.27)	__	76.49 (±1.29)	80.03 (±1.27)	__
	*S*. *neurona*	CV = 1.7%	CV = 1.6%		CV = 1.7%	CV = 2%	
	*N*. *caninum +*	78.6 (±1.70)	85.3 (±1.27)	__	78.85 (±1.38)	85.38 (±1.45)	__
	*C*. *parvum*	CV = 2.2%	CV = 1.55		CV = 1.7%	CV = 2%	
	*S*. *neurona +*	78.05 (±1.48)	80.55(±1.20)	__	77.85 (±1.45)	81.76 (±1.26)	__
	*C*. *parvum*	CV = 1.9%	CV = 1.5%		CV = 1.9%	CV = 2%	
Multiplex	*T*. *gondii* +	74.1 (± 1.27)	79.8 (±1.27)	__	75.9 (±1.48)	81.6 (±1.27)	__
	*N*. *caninum* +	CV = 1.7%	CV = 1.6%		CV = 2%	CV = 2%	
	S. neurona +						
	C. parvum						

^a^ Average between triplicates

^b^ Average between experiments; CV = Coefficient of variation.

The products of amplification with the primer pair described in this study were found to contain 35 polymorphic sites among the protozoa under study, as detected by the sequencing analysis. Three SNPs were found that differentiates *C*. *parvum* from *S*. *neurona*, and a single SNP was found that differentiates *N*. *caninum* from *T*. *gondii* (Table 2). The alignment in BLASTn revealed that the sequence of *S*. *neurona* shares 100% similarity with the *Sarcocystis neurona* TWC_2013 strain in the partial sequence of the 18S ribosomal RNA gene (gb KF854255.1), the sequence of *C*. *parvum* showed 92% similarity to *C*. *parvum* ribosomal RNA gene for 18S rRNA (pCPA931) (gb X64341.1), *T*. *gondii* showed 100% similarity with the *T*. *gondii* strain RH in the small-subunit ribosomal RNA gene (complete sequence; gb EF472967.1), and *N*. *caninum* showed 100% similarity with *N*. *caninum* 18S small subunit ribosomal RNA gene (partial sequence; gbGQ899206.1).

**Table 2:** Polymorphism sites of the amplicon rDNA 18S detected by DNA sequencing.

	Nucleotide position (fragment length ≅ 319 pb)																																		
Protozoan	25	47	92	93	109	136	175	176	183	184	186	187	188	191	192	189	195	209	210	211	213	216	217	218	219	221	222	226	234	257	259	260	285	287	298
*S*. *neurona*	G	T	A	A	G	A	C	G	A	C	G	C	T	C	A	G	A	A	T	G	T	C	A	C	C	A	T	G	C	A	A	A	G	T	G
(Strain 37R)																																			
G+C = 42.32%																																			
*C*. *parvum*	M	T	A	A	G	A	C	G	A	C	G	C	T	C	A	G	A	A	T	G	T	C	A	C	C	A	T	G	C	A	A	A	M	T	M
(Strain 13O)																																			
G+C = 41.07%																																			
*T*. *gondii*	G	C	G	G	A	G	A	A	G	A	A	A	A	-	-	A	C	T	G	G	G	-	-	-	-	T	C	A	T	-	T	G	G	A	G
(Strain RH)																																			
G+C = 42.31%																																			
*N*. *caninum*	G	C	G	G	A	G	A	A	G	A	A	A	A	-	-	A	C	T	G	A	G	-	-	-	-	T	C	A	T	-	T	G	G	A	G
(Strain NcBa)																																			
G+C = 41.99%																																			

M = A+C

The HRM analyses were performed using either genomic DNA samples of coccidia soon after the extraction of nucleic-acid samples or as the purified products of PCR of the 18S rDNA region for the same standardized amounts (gene copy numbers). The use of these two kinds of samples was intended to determine whether there was a discrepancy among the results because protozoa with more copies of this gene region may competitively inhibit the amplification of other protozoa’s genes (with a smaller number of copies) in the case of the direct use of genomic DNA. Competitive inhibition can occur when the relative concentration of a target is extremely high and competes for reagents and primers in a reaction [28]. This situation may affect the shape of the melting curves during the analysis, highlighting the protozoan curves with the largest number of copies instead of detecting those with fewer copies, thereby generating false negative results. Nevertheless, the curves produced in reactions based on genomic DNA were indistinguishable from curves produced in reactions using purified PCR products of 18S rDNA, having the same shapes and Tm peaks. That is, regardless of the differences among the copy numbers of this gene region, all amplification reactions yielded the same results as did PCRs of the samples in which the amounts of the DNA fragment of this region were normalized. This finding indicates that competitive inhibition did not occur during the amplification of these protozoa’s DNA, and that the genomic DNA may be used directly in a reaction to discriminate these species.

*T*. *gondii* strains RH (Type I), PTG (Type II), and CTG (Type III) showed derivative, normalized, and difference melting curves that were identical to one another. In the three genotypes, the melting curves showed a single peak (Tm = 79.85°C), and the coefficient of variation among and within experiments was less than 2.0% (Table 1), thus revealing high reproducibility. This result shows that the pair of primers used in this study amplifies a common region of *T*. *gondii* in the three genotypes, not differentiating among these strains; this method ensures specificity for species identification but not for genotyping. This feature can be considered an advantage when this method is used for interspecies discrimination (as in this study) because it is expected that parasites of the same species yield identical patterns of melting curves. Identification of different curves as a result of amplification of different genotypes of *T*. *gondii* can cause misdiagnosis and may lead to the assignment of each curve to a distinct species (and not to different genotypes of the same species). This is because the 18S rDNA does not provide information on intraspecific diversity. For *T*. *gondii*, the HRM method has shown good discrimination of genotypes by amplification of the B1 gene region in other studies. Costa et al. (2011) [25] compared the results of HRM with the results obtained by five microsatellite markers; HRM showed good discrimination of the major strains as compared to the microsatellite technique [25]. Askoy et al. (2014) [26] were able to detect and subtype *T*. *gondii* in samples of edible seafood, also using the B1 gene. Thus, the choice of the gene region to be detected must match the study purpose. A region with intraspecific genetic variability may not be suitable for detection of multiple pathogens but should be useful for the identification of multiple genotypes of the same species.

The *N*. *caninum* melting curve (Figs 1A, 2A, 2D and 2E) contained a single peak (Tm = 79.35°C) similar to that of *T*. *gondii* (Tm = 79.85°C; Table 1), with a difference of only 0.5°C, which is within the standard deviation. This difference can be detected because the acquisition of HRM fluorescence data is maximized due to dye saturation, which can detect differences as small as 0.01°C [18]. Because the Tm detection yielded very close peaks in the derivative melting curve, this type of graph ends up being uninformative for discrimination of very similar gene regions. In this case, the analysis of the normalized curve is more effective at revealing the differences among sequences [15]. Therefore, difference curves (Fig 1C) and normalized curves (Fig 2A) can clearly differentiate these protozoa. By sequencing, we detected only one SNP at position 211, (Table 2) differentiating the amplicon of *T*. *gondii* from that of *N*. *caninum*, where *T*. *gondii* DNA contains a guanine (…TG**G**AG…), whereas *N*. *caninum* DNA contains an adenine (…TG**A**AG…). Therefore, the derivative melting curves were very similar, showing a melting peak 0.5°C higher in *T*. *gondii* (which has the DNA site containing “G”) versus *N*. *caninum* (which has the DNA site containing “A”). Despite the difference between the amplified sequences in only one base pair, the HRM method showed a strong ability to discriminate these protozoans. It should be noted that *N*. *caninum* and *T*. *gondii* share many morphological and biological characteristics [29] that can confuse the diagnosis, depending on the technique used. They mostly have very similar genomes with conserved genetic features and a high degree of synteny, where most of the genes that encode proteins show one-to-one correspondence [1]. Due to this high degree of similarity, cross-reactive antigens between *N*. *caninum* and *T*. *gondii* may affect accuracy of diagnosis by immunofluorescence or an enzyme-linked immunosorbent assay (ELISA) [30]. In this regard, HRM has an advantage for differential diagnosis because it can clearly distinguish these species via the normalized (Figs 1B and 2A) and difference curves (Fig 1C).

Multiple Tm peaks were identified in *C*. *parvum* isolates. The derivative melting curves for *C*. *parvum* isolates showed three distinct peaks (Tm 1 = 76.65°C; Tm 2 = 80.7°C; Tm 3 = 85.15°C; Table 1) and all strains (13O, 13B, and 13F) yielded peaks identical to one another. As in *T*. *gondii*, the pair of primers used here could not detect intraspecific variability. Pangasa et al. (2009) [27] identified only one peak in the derivative melting curve for *C*. *parvum* (Tm = 72.17°C) by amplification of the ITS-2 target sequence in fecal samples suspected of cryptosporidiosis, in contrast to the three peaks identified in our study (Figs 1A, 2C, 2E and 2F). The use of different primers certainly may be responsible for the differences in the peaks because the shape of the peaks is related to G/C content, amplicon size, and gene sequences [15]. The coefficients of variation among and within experiments (Table 1) were lower than those obtained in the study by Pangasa et al. (2009) [27]. Thus, our data show high reproducibility of our method for the detection of *Cryptosporidium* by means of the 18S rDNA.

*S*. *neurona* yielded a derivative melting curve (Fig 1A) with two peaks (Tm 1 = 78.15°C; Tm 2 = 81.0°C), differentiating it from the other parasites. Three SNPs (present at positions 25, 285, and 298) were found to differentiate *S*. *neurona* from *C*. *parvum* (Table 2). Lalonde and Gajadhar (2011) [12], using a cocktail of seven primer pairs designed to amplify an 18S rDNA fragment, detected only one peak in the derivative melting curve of a species of *Sarcocystis* (Tm = 83.62°C); this approach allowed for the detection and discrimination of coccidia *Sarcocystis*, *Cryptosporidium*, *Eimeria*, *Isospora*, and *Toxoplasma*. However, these researchers used SYBR Green in their method as an intercalating agent for double-stranded DNA; this approach is less sensitive than saturation dyes used in the HRM analysis [31]. The presence of two peaks of Tm is mainly observed in heterozygous samples, where each peak represents an allele; there, SNPs can be easily identified due to changes in the shape of the curve [15, 32]. This is because the heteroduplex with higher G/C content (in this case *S*. *neurona* G + C = 42%) will show higher Tm than will the heteroduplex with higher A/C content (such as *C*. *parvum*: G + C = 41%). Multiple peaks may also be observed for repeated sequences (such as 18S rDNA). Accordingly, the difference in the stability between a hetero- and homoduplex is proportional to the degree of amplicon repetition in one allele; i.e., alleles with fewer repetitions have lower Tm, whereas alleles with an increased number of repetitions have higher Tm, yielding two Tm peaks in the derivative melting curve representing each allele [15]. Perhaps the shape of the curves between these two protozoans can also be explained because *C*. *parvum* has few repetitions of rDNA [33], whereas the genome of *S*. *neurona* is rich in repetitive sequences [34].

## Conclusions

Our results seem to be novel and show that the proposed method is sensitive and reproducible for identification and discrimination of these coccidia by 18S rDNA.

## Supporting information

S1 File Target rDNA 18S sequences (approximately 320 bp) containing conserved regions flanking sites with genetic variability among different coccidia, used for the primers design. Underlined sequences correspond to the foward and reverse primers.
(PDF)
