# Supplementary material for: Identification and discrimination of Toxoplasma gondii, Sarcocystis spp., Neospora spp., and Cryptosporidium spp. by righ-resolution melting analysis
Source: PLoS One. 2017 Mar 27;12(3):e0174168. doi: 10.1371/journal.pone.0174168 (PMC5367704; doi:10.1371/journal.pone.0174168)
Supplement: S1 File — Underlined sequences correspond to the foward and reverse primers. (PDF) [file pone.0174168.s001.pdf]

Supporting Information File 1: Target rDNA 18S sequences (approximately 320 bp) containing conserved regions flanking sites with genetic variability among different coccidia, used for the primers design. Underlined sequences correspond to the foward and reverse primers.

>*Toxoplasma gondii* strain RH (GenBank access number EF472967.1)

GTTGTTGCAGTTAAAAAGCTCGTAGTTGGATTTCTGCTGGAAGCAGCCAGTCCGCCCCAGGGGTGTGC
ACTTGGTGAATTCTAGCATCCTTCTGGATTTCTCCACACTTCATTGTGTGGAGTTTTTTCCAGGACTTTTA
CTTTGAGAAAATTAGAGTGTTTCAAGCAGGCTTGTCGCCTTGAATACTGCAGCATGGAATAATAAGATA
GGATTTCGGCCCTATTTTGTTGGTTTCTAGGACTGAAGTAATGATTAATAGGGACGGTTGGGGGCATTC
GTATTTAACTGTCAGAGGTGAAATTCTTAGAT

>*Neospora caninum* (GenBank access number U16159.1)

GTTGTTGCAGTTAAAAAGCTCGTAGTTGGATTTCTGCTGGAAGCAGCCAGTCCGCCCTCAGGGGTGTG
CACTTGGTGAATTCTAGCATCCTTCTGGATTTCTTCACACTTCATTGTGTGGAGTTTTTTCCAGGACTTTT
ACTTTGAGAAAATTAGAGTGTTTCAAGCAGGCTTGTCGCCTTGAATACTGCAGCATGGAATAATAAGAT
AGGATTTCGGCCCTATTTTGTTGGTTTCTAGGACTGAAGTAATGATTAATAGGGACGGTTGGGGGCATT
CGTATTTAACTGTCAGAGGTGAAATTCTTAGAT

>*Hammondia hammondi* (GenBank access number AF096498.1)

GTTGTTGCAGTTAAAAAGCTCGTAGTTGGATTTCTGCTGGAAGCAGCCAGTCCGCCCTCAGGGGTGTG
CACTTGGTGAATTCTAGCATCCTTCTGGATTTCTCCACACTTCATTGTGTGGAGTTTTTTCCAGGACTTTT
ACTTTGAGAAAATTAGAGTGTTTCAAGCAGGCTTGTCGCCTTGAATACTGCAGCATGGAATAATAAGAT
AGGATTTCGGCCCTATTTTGTTGGTTTCTAGGACTGAAGTAATGATTAATAGGGACGGTTGGGGGCATT
CGTATTTAACTGTCAGAGGTGAAATTCTTAGAT

>*Cryptosporidium parvum* (GenBank access number AF093494.1)

GTTGTTGCAGTTAAAAAGCTCGTAGTTGGATTTCTGTTAATAATTTATATAAAATATTTTGATGAATATTT
ATATAATATTAACATAATTCATATTACTATATATTTTAGTATATGAAATTTTACTTTGAGAAAATTAGAGT
GCTTAAAGCAGGCATATGCCTTGAATACTCCAGCATGGAATAATATTAAAGATTTTTATCTTTCTTATTG
GTTCTAAGATAAGAATAATGATTAATAGGGACAGTTGGGGGCATTTGTATTTAACAGTCAGAGGTGAA
ATTCTTAGAT

>*Cyclospora cayetanensis* (GenBank access number AF111183.1)

GTTGTTGCAGTTAAAAAGCTCGTAGTTGGATTTCTGTCGTGGTCATCCGGCCTTGCCCGTAGGGTGTGC
GCCTGGGTTGCCCGCGGCTTTCTTCCGGTAGCCTTCCGCGCTTCGCTGCGTGCGTTGGTGTTCCGGAAC
TTTTACTTTGAGAAAAATAGAGTGTTTCAAGCAGGCTTGTCGCCCTGAATACTGCAGCATGGAATAATA
AGATAGGACCTTGGTTCTATTTTGTTGGTTTCTAGGACCGAGGTAATGATTAATAGGGACAGTTGGGG
GCATTCGTATTTAACTGTCAGAGGTGAAATTCTTAGAT

>*Sarcocystis hominis* (GenBank access number AF176945)

GTTGTTGCAGTTAAAAAGCTCGTAGTTGGATATCTGCTGGAAGCAATCAGTCCGCCCTATTTTAGGGTG
TGCACTTGATGAATTCTGGCATCTATTATTCTTAATATAATGATTATTGAATTGATTTTCAATAATCAWTA
TTAGGAATAATACAGTTACTTTGAGAAAATTAGAGTGTTTGAAGCAGGCTTATTGCCTTGAATACTGCA

GCATGGAATAACAATATAGGATTTCGGTTCTATTATTTTGTTGGTTTGTAGGACTGAAATAATGATTAAT
AGGGACAGTTGGGGGCATTCGTATTTAACTGTCAGAGGTGAAATTCTTAGAT

>*Cystoisospora timoni* (GenBank access number AY279205.1)

GTTGTTGCAGTTAAAAAGCTCGTAGTTGGATTTCTGCTGGAAGCAGCCAGTCCGCCCTTAGGGGTGTGT
ACTTGGTGAAATTCCGGCATCCTCCTGGTAGCGCTTCACACTTAACTGGGTGGAGTGTTTTTCCAGGAC
TTTTACTTTGAGAAAATTAGAGTGTTTCAAGCAGGCTTGTTGCCTTGAATACTGCAGCATGGAATAATA
AGATAGGATTTCGGCCCTATTTTGTTGGTTTCTAGGACTGAAGTAATGATTAATAGGGACGGTTGGGG
GCATTCGTATTTAACTGTCAGAGGTGAAATTCTTAGAT

>*Nephroisospora eptesici* (GenBank access number EU334134.1)

GTTGTTGCAGTTAAAAAGCTCGTAGTTGGATTTCTGCTGGAAGCAGCCAGTCCGCCCTAAGGGGTGTG
CACTTGGTGAATTCTAGCATCCTTCTGGTGGTACTCCACACTTCGCTGTGTGGGGTGTTGTTCCAGGACT
TTTACTTTGAGAAAATTAGAGTGTTTCAAGCAGGCTTGTCGCCTTGAATACTGCAGCATGGAATAATAA
GATAGGATTTCGGCCCTATTTTGTTGGTTTCTAGGACTGAAGTAATGATTAATAGGGACGGTTGGGGG
CATTCGTATTTAACTGTCAGAGGTGAAATTCTTAGAT

>*Isospora belli* (GenBank access number AF106935.1)

GTTGTTGCAGTTAAAAAGCTCGTAGTTGGATTTCTGCTGGAAGCAGCCAGTCCGCCCTTAGGGGTGTGT
ACGTGGTGAAATTCCGGCATCCTTCTGGAGGAGCTTCGCACTTAACTGGGTGGAGTGCTTTTCCAGGAC
TTTTACTTTGAGAAAATTAGAGTGTTTCAAGCAGGCTTGTTGCCTTGAATACTGCAGCATGGAATAATA
AGATAGGATTTCGGCCCTATTTTGTTGGTTTCTAGGACTGAAGTAATGATTAATAGGGACGGTTGGGG
GCATTCGTATTTAACTGTCAGAGGTGAAATTCTTAGAT
